# Assessment of Macular Choroidal Thickness via Enhanced Depth Imaging Optical Coherence Tomography in Patients With Chronic Tinnitus

**DOI:** 10.1155/joph/4062675

**Published:** 2026-04-07

**Authors:** Yu-Chi Sung, Yo-Chen Chang, Wei-Lun Chu, Kuo-Jen Chen, Kai-Chun Cheng

**Affiliations:** ^1^ Department of Ophthalmology, Kaohsiung Medical University Hospital, Kaohsiung, Taiwan, kmuh.org.tw; ^2^ Department of Ophthalmology, School of Medicine, College of Medicine, Kaohsiung Medical University, Kaohsiung, Taiwan, kmu.edu.tw; ^3^ Department of Ophthalmology, Kaohsiung Municipal Siaogang Hospital, Kaohsiung, Taiwan, kmu.edu.tw; ^4^ Department of General Medicine, Kaohsiung Medical University Hospital, Kaohsiung, Taiwan, kmuh.org.tw

**Keywords:** choroidal thickness, chronic tinnitus, EDI-OCT

## Abstract

**Purpose:**

This study investigates the relationship between macular choroidal thickness (CT) and chronic tinnitus using enhanced depth imaging optical coherence tomography (EDI‐OCT).

**Study Design:**

This retrospective study was conducted between January 2018 and October 2022.

**Methods:**

A total of 372 eyes from 338 patients were analyzed, divided into tinnitus (127 eyes) and control (245 eyes) groups. Macular CT was measured subfoveally and 2 mm around the central fovea.

**Results:**

Macular CT showed significant thinning in the tinnitus group (156.4 ± 59.8 μm versus 240.3 ± 71.0 μm, *p* < 0.01). Specifically, subfoveal CT (SFCT) was thinner in all directions—temporal, nasal, superior, and inferior to the central fovea. CT decreased with advanced age in both groups, with the tinnitus group consistently exhibiting significantly thinner choroid compared to controls within the same age range (*p* < 0.05). In the control group, there was a notable correlation between myopia and thinner SFCT (*p* < 0.01), whereas no significant difference was observed in the tinnitus group (*p* = 0.984).

**Conclusion:**

Our study utilized EDI‐OCT as a precise tool to measure macular CT, revealing significantly thinner CT in tinnitus patients. These findings suggest that disturbances in choroidal perfusion may be related to chronic tinnitus, highlighting CT as a potential biomarker.

## 1. Introduction

The choroid, located in the posterior segment of the uveal tract, is a thin but highly vascularized layer. Situated between the retina and the sclera, it provides a rich and steady blood supply to the outer retina, retinal pigment epithelium (RPE), the foveal avascular zone, and the optic nerve [1]. The physiological functions of the choroid include supporting the normal metabolism of the RPE and photoreceptors, regulating the thermal environment of the retina, facilitating the removal of metabolic byproducts, and secreting growth factors [2, 3].

Optical coherence tomography (OCT) serves as a crucial noninvasive imaging technique in clinical ophthalmology to obtain high‐resolution images of both the retina and choroid [4]. OCT allows visualization of interlayer relationships and provides morphological insights into retinal‐choroidal diseases. Choroidal thickness (CT) refers to the distance measured from the outer edge of the hyperreflective line representing the RPE to the choroidoscleral interface in OCT images. Enhanced depth imaging‐OCT (EDI‐OCT) is an effective technique for accurately measuring CT.

By positioning the zero‐delay line closer to the choroid, EDI‐OCT improves image resolution and facilitates clearer identification of the choroidoscleral interface. Additionally, the use of image averaging techniques increases the signal‐to‐noise ratio, resulting in clearer images with reduced speckle noise, which further enhances choroid visualization [5]. These benefits collectively make EDI‐OCT an effective tool for accurately measuring CT.

CT can serve as a sensitive biomarker for predicting, diagnosing, intervening, and monitoring various ocular diseases. SFCT typically ranges from 191 ± 74.2 to 354 ± 111 microns [6–8]. Measurement variability can be influenced by factors such as ethnicity, gender, age, refractive error, axial length, and diurnal changes. Notably, CT varies significantly between the subfoveal area and all extrafoveal locations, with the greatest thickness observed directly beneath the fovea. Thickness decreases rapidly in both nasal and temporal directions away from the fovea, and the choroid is thinner in the inferior macular region compared to the superior macular region [9]. Thickening of CT can result from choroidal vasodilation or hyperperfusion, leading to increased choroidal permeability and focal choroidal exudation observed in conditions such as central serous chorioretinopathy (CSCR) [10], polypoidal choroidal vasculopathy (PCV) [11], and active Vogt–Koyanagi–Harada (VKH) disease [12]. Conversely, hypoperfusion and choroidal thinning are associated with metabolic disorders of the RPE and photoreceptors, observed in age‐related macular degeneration (AMD) [13], pathological myopia [14], idiopathic macular hole [15], hereditary retinal dystrophies [16], and long‐standing VKH diseases [17]. CT alterations can also be observed in systemic diseases involving vascular components, such as chronic kidney disease [18], cardiovascular diseases [19], systemic autoimmune syndromes [20], and Alzheimer’s disease [21]. These findings suggest that CT may reflect not only ocular microvascular changes but also systemic vascular dysfunction.

Subjective tinnitus manifests as the perception of sound without any external stimulus, affecting 5%–42% of the general population chronically [22]. Previous studies have established the association between tinnitus and irregular neuronal activity [23]. However, the exact mechanisms behind abnormal neuronal firing in tinnitus remain under investigation. Limited insights exist regarding the correlation between CT and chronic tinnitus. In 2008, C. Erb et al. used ultrasound to measure CT and found a notable increase among chronic tinnitus patients without ophthalmological pathological findings (150 ± 20 versus 120 ± 15 μm, *p* = 0.033) [24].

To date, no study has comprehensively evaluated CT in chronic tinnitus using EDI‐OCT, which provides more reliable and reproducible measurements than ultrasound. Therefore, the present study aimed to investigate the relationship between macular CT and chronic subjective tinnitus using EDI‐OCT and to explore whether changes in CT may serve as a potential ocular biomarker reflecting microvascular alterations associated with tinnitus.

Understanding this relationship could provide novel insights into the vascular component of tinnitus pathophysiology and support the potential role of ocular imaging as a noninvasive tool for systemic disease evaluation.

## 2. Methods

### 2.1. Subjects Recruitment

A total of 372 eyes from 338 patients were retrospectively recruited from January 2018 to October 2022 at Kaohsiung Medical University Hospital, a tertiary care center in Taiwan. All the patients received comprehensive ophthalmic examinations at the ophthalmology clinic, including best‐corrected visual acuity (BCVA), intraocular pressure measurement (IOP), slit‐lamp biomicroscopy to evaluate the anterior segment for any abnormalities, dilated fundus examination, EDI‐OCT imaging, assessment of disc morphology, and confirmation of the patient’s refractive status. OCT scans were performed between 9:00 a.m. and 1:00 p.m., and because the examinations were conducted during morning clinic hours, the majority of patients underwent imaging within this time window, minimizing the effect of diurnal variation and ensuring consistency across participants [25]. This study received approval from the institutional review board of Kaohsiung Medical University Hospital (KMUHIRB‐E(I)‐20230,136) and was conducted in accordance with the ethical standards of the Declaration of Helsinki. Due to the retrospective nature of the study, informed consent was waived.

Patients were included based on the following criteria: (1) age ≥ 20 years, (2) BCVA of 20/30 or better, (3) refractive error less than −6.5 *D* of myopia or less than +3.0 *D* of hyperopia or 3.0 *D* of cylinder, and (4) diabetes mellitus without diabetic retinopathy. Exclusion criteria encompassed: (1) history of myopia exceeding −6.5 *D*, (2) amblyopia, (3) any type of glaucoma, (4) any type of retinopathy, (5) epiretinal membrane, (6) history of retinal detachment, (7) uveitis, (8) ocular trauma, (9) ocular tumor, (10) history of macular surgery, (11) AMD, (12) CSCR, (13) choroidal neovascularization, (14) drusen larger than 125 μm or confluent drusen, (15) retinovascular abnormalities, (16) intravitreal medications, and (17) poor image quality with a signal strength index (SSI) below 7/10 or with poorly visible choroidoscleral interfaces or foveal contour.

### 2.2. Tinnitus Group and Control Group

The participants were divided into two groups in this study. The tinnitus group included patients diagnosed with chronic tinnitus (ICD‐9388.3, ICD‐10 H93.3) by otolaryngologists at Kaohsiung Medical University Hospital. The diagnosis of tinnitus was confirmed through comprehensive audiological assessments, including pure‐tone audiometry, conducted at the hospital’s otolaryngology clinic. Participants presented with a variety of underlying causes of tinnitus, such as noise exposure, age‐related hearing loss, and cases where the etiology remained unknown. The control group comprised individuals who did not have tinnitus or any other hearing impairments.

### 2.3. Measurements

**FIGURE 1:**
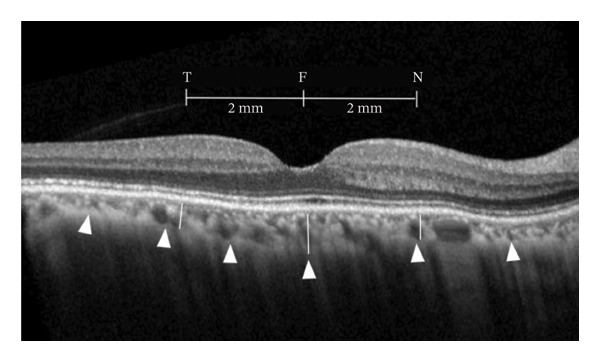
Choroidal measurement method in our study. Choroidal thickness measurements were taken at the central fovea and 2 mm from the temporal, nasal, superior, and inferior regions relative to the central fovea and defined as the distance between the outer border of the RPE‐Bruch’s membrane complex and the choroidoscleral border. ^∗^F = fovea; N = nasal; T = temporal.

In this study, CT was measured using EDI‐OCT (Spectralis, Heidelberg Engineering, Germany). Demographic data including age, gender, and spherical equivalent refractive error (SERE) were stratified and recorded. Myopia was defined as SERE < 0.00. CT measurements were taken at the central fovea and 2 mm from the temporal, nasal, superior, and inferior regions relative to the central fovea. CT was defined as the distance between the outer border of the RPE‐Bruch’s membrane complex and the choroidoscleral border (Figure 1).

### 2.4. Statistical Analysis

The results are presented as mean ± standard error of the mean with a 95% confidence interval. The normality of the data distribution in each group was assessed using the Shapiro–Wilk test and visual inspection of quantile–quantile (Q–Q) plots. For normally distributed variables, group comparisons were performed using independent‐samples *t*‐tests; otherwise, the Mann–Whitney *U* test was applied. Subgroup comparisons within the group (e.g., myopic vs. nonmyopic) followed the same criteria. In addition to *p* values, effect sizes were calculated to quantify the magnitude of between‐group differences. A two‐sided *p* value < 0.05 was considered statistically significant. Statistical analyses were conducted using SPSS version 27.0 (SPSS Inc., Chicago, IL, USA).

In healthy adults aged over 60 years, the normal subfoveal CT (SFCT) typically ranges from approximately 220–260 μm [26, 27]. Based on this reference range, we assumed a mean difference of 30 μm in SFCT between the tinnitus group and healthy controls, with an estimated standard deviation of 70 μm, corresponding to a moderate effect size (Cohen’s *d* = 0.43). Using a two‐sided independent‐samples *t*‐test with a significance level of *α* = 0.05, a total of 174 participants (87 per group) would be required to achieve 80% statistical power, as calculated using GPower version 3.1.9.7.

## 3. Results

The demographic characteristics are detailed in Table 1, comparing 127 eyes from 93 subjects in the tinnitus group to 245 eyes from 245 subjects in the control group. The mean age of participants in the tinnitus group was 63.5 ± 13.2 years, notably higher than the 57.3 ± 12.3 years observed in the control group. The age distribution is categorized, revealing a higher percentage of elderly individuals in the tinnitus group. Gender distribution was similar between the two groups, with 67.7% females and 32.3% males in the tinnitus group, compared to 68.6% females and 31.4% males in the control group. The mean SERE was −1.56 ± 2.67 in the tinnitus group and −1.36 ± 2.50 in the control group. Although a slight difference in refractive error between the two groups was observed, it was not statistically significant (*p* = 0.649). In our study, myopia was defined as SERE < 0, with a prevalence of 55.9% in the tinnitus group and 50.2% in the control group (Table 2).

**TABLE 1:** Patients characteristic of both groups.

	**Tinnitus group (*n* = 93)**	**Control group (*n* = 245)**

Age (years)	63.52 ± 13.2	57.3 ± 12.3
< 40	7 (7.5)	31 (12.7)
41–50	8 (8.6)	36 (14.6)
51–60	12 (12.9)	72 (29.4)
61–70	36 (38.7)	75 (30.6)
> 71	30 (32.3)	31 (12.7)
Gender		
Male	30 (32.3)	77 (31.4)
Female	63 (67.7)	168 (68.6)

**TABLE 2:** Refraction status and subfoveal choroidal thickness of both groups.

	Tinnitus group (*n* = 127)	Control group (*n* = 245)	*p* value
SERE (Diopter)	−1.56 ± 2.67	−1.36 ± 2.50	0.649
Without myopia	56 (44.1)	122 (49.8)	
With myopia	71 (55.9)	123 (50.2)	
SFCT (μm)	156.4 ± 59.8	240.3 ± 71.0	< 0.01
Nasal side (μm)	142.2 ± 59.8	188.1 ± 84.0	< 0.01
Temporal side (μm)	170.9 ± 59.0	241.2 ± 70.8	< 0.01
Superior side (μm)	174.0 ± 65.3	231.7 ± 81.9	< 0.01
Inferior side (μm)	154.0 ± 63.1	269.2 ± 75.1	< 0.01

*Note:* SFCT, subfoveal choroidal thickness.

Abbreviation: SERE, spherical equivalent refractive error.

CT was measured in all recruited subjects using EDI‐OCT images by the same experienced clinician. The overall SFCT was 156.4 ± 59.8 μm in the tinnitus group and 240.3 ± 71.0 μm in the control group. The mean between‐group difference was −83.9 μm (95% confidence interval [CI]: −92.5 to −75.3 μm), corresponding to a large effect size (Cohen’s *d* = 1.25), indicating significantly decreased CT in the tinnitus group (*p* < 0.01). We additionally assessed various choroidal regions (2 mm from the central fovea in nasal, temporal, superior, and inferior directions) and found thinner measurements in all regions in the tinnitus group compared to the control group. Statistical analysis revealed significant differences in each region, with *p* values < 0.01 (Table 2).

To examine the correlation between age and CT, subjects in both groups were categorized into age brackets, as shown in Table 3. There was a noticeable decline in CT with advancing age in both groups across all measured directions. In the tinnitus group, measurements ranged from 194.6 ± 49.4 μm in the youngest age group to 145.4 ± 66.0 μm in the oldest age group in the central foveal area. In the control group, measurements ranged from 255.1 ± 39.2 μm in the youngest age group to 229.2 ± 98.9 μm in the oldest age group in the central foveal area. Our data revealed significantly thinner SFCT in the tinnitus group across all age groups, with *p* values < 0.05 in each group.

**TABLE 3:** Age correlation of both groups.

Age (years)	Choroid thickness (μm)	Nasal side (μm)	Temporal side (μm)	Superior side (μm)	Inferior side (μm)
Tinnitus group					
≤ 40	194.6 ± 49.4	190.6 ± 53.6	204.9 ± 49.7	214.9 ± 25.7	196.3 ± 39.7
41–50	181.3 ± 88.3	159.7 ± 84.3	203.2 ± 74.4	197.8 ± 68.3	199.3 ± 68.3
51–60	155.6 ± 33.7	137.6 ± 36.5	166.6 ± 36.4	171.6 ± 41.2	157.1 ± 45.8
61–70	152.4 ± 51.9	136.2 ± 48.7	166.1 ± 54.8	174.3 ± 60.4	145.3 ± 59.0
≥ 71	145.4 ± 66.0	135.6 ± 69.1	161.1 ± 64.5	157.7 ± 79.1	140.8 ± 68.9
Control Group					
≤ 40	255.1 ± 39.2	201.2 ± 76.0	270.3 ± 56.6	232.3 ± 77.4	286.3 ± 56.9
41–50	252.0 ± 53.1	210.8 ± 73.2	253.3 ± 58.0	256.2 ± 67.7	283.1 ± 67.9
51–60	240.6 ± 87.7	204.9 ± 86.8	236.0 ± 76.5	248.6 ± 81.8	265.1 ± 80.5
61–70	232.8 ± 55.0	162.6 ± 69.9	236.9 ± 58.6	216.5 ± 69.8	270.6 ± 67.5
≥ 71	229.2 ± 98.9	171.5 ± 109.7	220.3 ± 98.8	199.9 ± 111.3	242.4 ± 98.6

Previous research indicates that myopic eyes often have thinner choroids compared to nonmyopic eyes [28]. To minimize the impact of myopia, we analyzed the correlation between myopia and CT within the tinnitus and control groups, respectively. Table 4 shows that within the tinnitus group, there were no statistically significant differences in CT between myopic and nonmyopic individuals across all five regions examined, with *p* values > 0.05 for each region. In contrast, Table 5 highlights significant differences in the control group, where myopic individuals exhibited thinner choroidal measurements than their nonmyopic counterparts across all regions, with *p* values < 0.01.

**TABLE 4:** Correlation in myopic cases of tinnitus group.

	Myopia group (*n* = 71)	No myopia group (*n* = 56)	**p** value
Choroid thickness (μm)	156.5 ± 61.4	156.2 ± 68.2	0.984
Nasal side (μm)	142.7 ± 61.3	141.6 ± 58.5	0.903
Temporal side (μm)	174.4 ± 58.2	166.3 ± 60.1	0.449
Superior side (μm)	178.8 ± 61.8	167.9 ± 69.4	0.362
Inferior side (μm)	157.6 ± 62.5	149.4 ± 64.2	0.470

**TABLE 5:** Correlation in myopic cases of control group.

	Myopia group (*n* = 123)	No myopia group (*n* = 122)	**p** value
Choroid thickness (μm)	228.0 ± 57.7	252.7 ± 80.5	< 0.01
Nasal side (μm)	168.4 ± 60.9	208.0 ± 98.4	< 0.01
Temporal side (μm)	227.9 ± 62.1	254.6 ± 76.6	< 0.01
Superior side (μm)	212.9 ± 65.3	250.6 ± 92.3	< 0.01
Inferior side (μm)	250.8 ± 66.9	287.8 ± 79.1	< 0.01

## 4. Discussion

In this study, we investigated the relationship between macular CT and chronic tinnitus, specifically examining the effects of age and myopia within this patient cohort. Our analysis revealed no statistically significant differences in the distribution of these variables between the groups, effectively isolating the specific impact of tinnitus on CT. The findings demonstrated that CT is generally reduced in tinnitus patients compared to normal individuals, with the most significant differences observed in the fovea and inferior regions, while variations were less marked in the nasal region. Interestingly, our findings contrast to the results of C. Erb et al. [24], who reported increased CT in chronic tinnitus patients. We attribute these differences to the larger sample size in our study and the more precise measurement tool of EDI‐OCT compared to ultrasonography. Despite yielding different results, both our study and Erb et al. suggest that choroidal perfusion disturbance could be indicative of chronic tinnitus in patients.

The relationship between tinnitus and decreased CT remains incompletely understood. One hypothesis is that tinnitus‐related choroidal thinning could result from ischemia affecting choroidal blood vessels, leading to reduced perfusion [29]. Further research is needed to determine whether this phenomenon is a causal factor, an effect, or if both stem from a shared underlying cause. Previous studies have suggested that the amygdalohippocampal complex, which receives blood supply from the anterior choroidal artery, may influence tinnitus symptoms [30]. The anterior choroidal artery, originating from the internal carotid artery along with the ophthalmic artery, could be affected by hypoperfusion in the internal carotid artery due to specific conditions, leading to both a decrease in CT and the onset of tinnitus. Such conditions may include systemic diseases such as chronic kidney disease [18, 31], cardiovascular disease [19, 32], and autoimmune disease [20, 33].

There are various factors that can influence CT. Previous research has established a negative correlation between age and CT, highlighting aging as a critical determinant of choroidal thinning. Studies have reported that the average reduction in SFCT is 2.77–2.98 μm per year [34, 35]. This vascular decline of the choroid in older individuals might compromise its capacity to supply sufficient oxygen and other metabolites to the RPE and outer retina, leading to structural thinning of the choroid. Similar trends were observed in subjects with chronic tinnitus in our study. This correlation underscores the progressive nature of structural changes in the eye associated with aging and tinnitus, potentially emphasizing the combined effects of chronic tinnitus and age‐related ocular modifications.

In contrast to aging, previous studies have identified a positive correlation between myopia and CT, suggesting that myopia may elongate the axial length of the eye, potentially affecting retinal function [36]. This indicates that SERE might be a significant factor in choroidal thinning, as shown in Table 4. However, our study found that the tinnitus patient group did not exhibit the same pattern, with no statistically significant differences in CT between myopic and nonmyopic individuals across all examined regions. A comparative analysis of Tables 3 and 4 reveals a distinct interaction between tinnitus and myopia regarding CT. While myopia significantly affects CT in the general population, its impact appears to be attenuated in individuals with tinnitus, suggesting that tinnitus and myopia may share underlying mechanisms contributing to choroidal thinning, with tinnitus exerting a more pronounced effect.

To our knowledge, this study represents the largest cohort investigating the correlation between chronic tinnitus and CT. There are several limitations to this study. Firstly, it is a retrospective review, and not all subjects underwent EDI‐OCT during their initial examination. Some cases with inadequate choroidal imaging on SD‐OCT were excluded from the study, potentially resulting in an underestimation of eligible cases. Secondly, fewer patients were recruited in the tinnitus group compared to the control group, which could introduce bias. Thirdly, axial length measurements were not available for all participants due to the retrospective nature of the study. However, patients with high myopia greater than 6.5 diopters or with other significant refractive errors that could influence CT were excluded to minimize the effect of axial length variation. Finally, CT measurements were manually performed, which may introduce inter‐ and intra‐observer variability, as noted in previous studies [37].

In conclusion, our study utilized the highly precise measurement tool, EDI‐OCT, to assess CT in both the chronic tinnitus group and normal individuals, revealing different results from a previous study. Our findings support the hypothesis of thinner CT in tinnitus patients, possibly associated with choroidal perfusion disturbance or ischemia. We also confirmed that age has a significant negative correlation with CT in patients with tinnitus, consistent with previous research in the general population. Additionally, the changes in CT among subjects with both tinnitus and myopia were less pronounced than those in subjects with myopia alone. The analysis also indicated that CT tended to be thinner on the nasal side compared to the temporal side, regardless of age and myopia subgrouping. We look forward to future studies that will further elucidate the pathogenesis linking chronic tinnitus and choroidal thinning and explore the potential of CT as a biomarker for chronic tinnitus.

## Funding

This study was financially supported by grants KMUH112‐2R49, KMUH113‐3R46, and KMUH114‐4R51 from Kaohsiung Medical University Hospital, Taiwan, and Grants 113‐2320‐B‐037‐013 and 114‐2320‐B‐037‐007‐MY2 from the National Science and Technology Council, Taiwan.

## Conflicts of Interest

The authors declare no conflicts of interest.

## Data Availability

Research data are not shared.
